# Cardiac-specific overexpression of PRMT5 exacerbates pressure overload-induced hypertrophy and heart failure

**DOI:** 10.1186/s12929-025-01162-6

**Published:** 2025-07-06

**Authors:** Yasufumi Katanasaka, Yoichi Sunagawa, Ryoga Sakurai, Mikuto Tojima, Ryuya Naruta, Yuya Hojo, Yuto Kawase, Toshihide Hamabe-Horiike, Kiyoshi Mori, Koji Hasegawa, Tatsuya Morimoto

**Affiliations:** 1 Division of Molecular Medicine, School of Pharmaceutical Sciences, University of Shizuoka, 52-1 Yada, Suruga-ku, Shizuoka, 422-8526 Japan; 2 Division of Translational Research, National Hospital Organization Kyoto Medical Center, 1-1 Mukaihata-cho Fukakusa, Fushimi-ku, Kyoto, 612-8555 Japan; 3 Shizuoka General Hospital, 4-27-1 Kita Ando Aoi-ku, Shizuoka, 420-8527 Japan; 4 Graduate School of Public Health, Shizuoka Graduate University of Public Health, Shizuoka, 4200881 Japan; 5 Department of Molecular and Clinical Pharmacology, School of Pharmaceutical Sciences, University of Shizuoka, Shizuoka, 4228526 Japan

**Keywords:** Protein arginine methyltransferase 5, Cardiac hypertrophy, Heart failure, Arginine methylation, p300, Histone acetyltransferase

## Abstract

**Background:**

Various epigenetic modifiers are involved in the regulation of gene expression during pathological cardiac hypertrophy—a critical event in the development of heart failure. Our previous research has demonstrated that protein arginine methyltransferase 5 (PRMT5) in cardiac fibroblasts is a crucial epigenetic writer implicated in pathological cardiac fibrosis. Moreover, treatment with a PRMT5 inhibitor also suppressed cardiac hypertrophy in mice after transverse aortic constriction (TAC) surgery. However, as the functional role of PRMT5 in cardiomyocytes remains to be fully elucidated in pathological cardiac hypertrophy and systolic dysfunction, this study aimed to clarify the gain-of-function of PRMT5 in cardiomyocytes.

**Methods:**

Cardiac-specific PRMT5 transgenic (PRMT5-TG) mice were generated to evaluate the gain-of-function of PRMT5 in cardiac hypertrophy and dysfunction in male mice undergoing TAC surgery. Cardiac function and myocardial cell hypertrophy were evaluated in wild-type (WT) and PRMT5-TG mice after TAC surgery. To elucidate the molecular mechanistic basis through which PRMT5 induces cardiomyocyte hypertrophy, we examined epigenetic modifications of histones in cardiomyocytes.

**Results:**

Echocardiography revealed that fractional shortening was reduced in PRMT5-TG mice compared to WT mice after TAC surgery. Both heart weight/BW and lung weight/BW ratios increased significantly more in PRMT5-TG than in WT mice. Histological analyses showed that cardiomyocyte diameter and perivascular fibrosis were elevated in PRMT5-TG mice in comparison to WT mice. Hypertrophic gene expression significantly increased in PRMT5-TG mice after TAC surgery. In primary cultured neonatal rat cardiac myocytes, EPZ015666, a specific inhibitor of PRMT5, and PRMT5 knockdown significantly inhibited phenylephrine (PE)-induced cell hypertrophy. Cardiac overexpression of PRMT5 promoted the acetylation of H3K9, a histone marker associated with cardiomyocyte hypertrophy, and the histone acetyltransferase activity of p300. Conversely, treatment with EPZ015666 reduced the acetylation of H3K9 induced by TAC surgery and PE treatment. Finally, we found that PRMT5 interacts with and methylates p300 at R200. The R200 point mutation in p300 abolished PRMT5-mediated enhancement of its histone acetyltransferase activity.

**Conclusions:**

The gain-of-function of PRMT5 in cardiomyocytes exacerbates pressure overload-induced cardiac hypertrophy and left ventricular systolic dysfunction, at least partially, through p300 methylation and histone acetyltransferase activation.

**Supplementary Information:**

The online version contains supplementary material available at 10.1186/s12929-025-01162-6.

## Background

The global prevalence of heart failure is predicted to increase. Given the significant influence of heart failure on morbidity, mortality, and healthcare expenditure, improvements in therapy are needed [1, 2]. The adaptive mechanism of the heart in maintaining contractility and function involves cardiac hypertrophy. However, persistent cardiac stress from pathological conditions, including valvular disease, myocardial infarction, and hypertension, can contribute to detrimental pathological hypertrophy, ultimately resulting in heart failure [3]. The inhibition of pathological myocardial hypertrophy would be a successful strategy for heart failure pharmacotherapy [4]. Ongoing research aims to identify molecular targets for the suppression of left ventricular hypertrophy.

During the pathological progression of myocardial hypertrophy, various alterations in cardiomyocytes have been observed, such as an increase in protein synthesis and changes in fetal gene expression [5]. The expression of fetal genes, including *Myh7*, *Nppa*, and *Nppb*, is regulated by several transcription factors and transcriptional co-factors in cardiomyocytes [6–8]. Recent studies have enhanced our understanding of the epigenetic regulatory mechanisms of gene transcription, including post-translational modification of histones and DNA methylation, during the development and progression of cardiac failure [9, 10]. Studies have shown that certain compounds inhibiting histone acetylation and methylation may positively influence cardiac remodeling and function [7]. Among the epigenetic regulators, we have investigated the function of p300, a transcriptional co-activator, in the progression and development of myocardial cell hypertrophy and left ventricular dysfunction [6, 9, 11]. The p300 function as a histone acetyltransferase (HAT) plays a crucial role in heart failure progression through the acetylation of histones and a variety of transcription factors [6, 12]. Histone acetylation, such as H3K9 acetylation, is reported to be essential for cardiomyocyte hypertrophy [13]. Moreover, p300 HAT activity inhibitors attenuate cardiac cell hypertrophy and prevent cardiac failure development in vivo [9, 14, 15]. Thus, epigenetic regulators may serve as molecular targets for anti-hypertrophic therapies.

A prevalent post-translational modification is the methylation of arginine, with nuclear proteins in cells containing approximately 0.5–2% methylated arginine residues [16]. Protein arginine methyltransferases (PRMTs) are ubiquitously expressed in various cells and have been implicated in a variety of biological processes, such as cell proliferation and differentiation [17]. PRMT5, a type II arginine methyltransferase, catalyzes the symmetrical dimethylation of arginine residues within proteins, particularly within glycine-arginine-rich (GAR) motifs [18]. Histone arginine methylation by PRMT5 has been reported to control both transcriptional activation and repression. H3R2me2s correlates with the activation of gene transcription, whereas symmetrical dimethylation of H4R3 and H3R8 represses a set of gene transcriptions [19]. PRMT5 is also associated with multiple proteins, such as kinases, transcription factors, and transcriptional co-factors, and regulates multiple biological processes [19]. PRMT5 dysregulation contributes to the pathogenesis of several diseases, including cancer, inflammation, and metabolic disorders [20–22].

The functions of PRMT5 in cardiomyocytes have been previously reported [23–25]. These reports have shown that endogenous PRMT5 in cultured cardiomyocytes protects against cardiac hypertrophy. Additionally, Li et al. reported that the conditional knockout of *Prmt5* in cardiomyocytes induces dilated cardiomyopathy [26], suggesting that endogenous PRMT5 in cardiac myocytes is essential to maintain the homeostasis of cardiac metabolism. In contrast, our previous study showed that treatment with a PRMT5 inhibitor significantly suppressed pressure overload-induced pathological cardiac hypertrophy, myocardial fibrosis, and left ventricular systolic dysfunction [27]. Therefore, we believe that the function of PRMT5 in cardiomyocytes under physiological and pathological conditions remains controversial. In some specific pathological conditions, PRMT5 is upregulated and activates downstream signaling pathways. For example, the gain-of-function of PRMT5 has been demonstrated to contribute to cancer progression, with its regulatory influence extending to various signaling pathways [19]. However, the gain-of-function of PRMT5 in pathological cardiac hypertrophy in vivo remains unknown. The objective of this study was to elucidate the function and mechanism of action of PRMT5 in cardiomyocytes using a cell culture study and an animal model of heart failure induced by pressure overload.

To clarify the gain-of-function of PRMT5 during myocardial hypertrophy, transgenic mice that specifically expressed PRMT5 in cardiomyocytes were generated. The current study provides evidence that PRMT5 interacts with p300, regulates p300 HAT activity, and promotes pressure overload-induced cardiomyocyte hypertrophy. Our results reveal a novel molecular mechanism by which PRMT5 modulates pathological cardiac hypertrophy through histone acetylation.

## Methods

### Materials

The pcDNA3-Myc plasmid vectors encoding human PRMT5 and PRMT5 point mutant (G367A), which lacks methyltransferase activity, were kindly provided by Dr. Naoya Fujita (Japanese Foundation for Cancer Research) [28]. The pCMV-p300 expression vector has been previously described [11]. The pGEX6P1 vector (GE Healthcare, Buckinghamshire, UK) was used as the target for insertion of the PCR-amplified p300 mutant sequences. For lentivirus transduction, FLAG-tagged human PRMT5 was subcloned to a CSII-CMV backbone plasmid vector using restriction enzymes. EPZ015666 and C646 were supplied by DC Chemicals (Shanghai, China) and Sigma-Aldrich (Tokyo, Japan), respectively. A485 was purchased from MedChemExpress (Monmouth Junction, NJ, USA). Small interfering RNAs were obtained–Mission siRNA (Universal Negative Control for rat PRMT5 [SASI_Rn02_00242865]) (Sigma-Aldrich).

### Mice

PRMT5 transgenic mice were generated by the RIKEN BioResource Research Center (Tsukuba, Japan). Briefly, the *Prmt5* plasmid vector was assembled by subcloning the *Prmt5* fragment into the *SalI*-*NheI* site of a plasmid vector containing the mouse α-myosin heavy chain (MHC) promoter, generously gifted by Dr. Sanbe (Iwate Medical University) as previously described [29]. C57BL/6j male mice (8 weeks of age) were acquired from Japan SLC Corporation (Hamamatsu, Japan). The animals were housed in a dedicated pathogen-free facility with a 12-h light/dark schedule. They also had unrestricted access to food and water. The total number of mice used in each experiment in this study is given in the figures or the corresponding legends. All study procedures were approved by the Institutional Animal Care and Use Committee (IACUC) of the University of Shizuoka (#186316) and NHO Kyoto Medical Center (#2-25-3).

### Cell culture

The method for isolating primary cardiomyocytes was adapted from a previously described protocol with some adjustments [30]. The hearts from SD rats (1-day-old) were dissected into small pieces. They were incubated at 37 °C in a digestion solution containing collagenase type II (#CLS2, Worthington Industries, Worthington, OH, USA) and pancreatin (Sigma-Aldrich) in Hank’s balanced salt solution (HBSS, Nacalai Tesque, Kyoto, Japan). Both primary cardiomyocytes and HEK293T cells (RIKEN BioResource Center, Tsukuba, Ibaraki, Japan) were cultured in Dulbecco’s modified Eagle medium (DMEM, Nacalai Tesque) with the supplement of the antibiotics (penicillin/streptomycin) and 10% heat-inactivated FBS. Cells were maintained at 37 °C under a 5% CO_2_ atmosphere in a humidified chamber. Using Lipofectamine LTX (Thermo Fisher Scientific, Tokyo, Japan), expression vectors containing cDNA for PRMT5 or p300 were transfected into cells following the protocol of the manufacturer [8]. Lentiviral transduction to neonatal cardiomyocytes was performed as described previously [31].

### Transverse aortic constriction (TAC)

The TAC surgery procedure was executed as outlined in previous research [30]. Male mice (aged 8–10 weeks) were subjected to anesthesia via 2% isoflurane inhalation. To access the chest cavity, an incision was made in the upper portion of the sternum. The transverse thoracic aorta was then constricted by tying it off between the innominate artery and left common carotid artery, employing a 27-gauge blunt needle and 6–0 silk suture. Following surgery, all mice received buprenorphine for 2 consecutive days. Control mice underwent sham operations without aortic constriction. To ensure impartiality, a researcher unaware of the mice’s genotypes performed the surgeries for each group.

### Echocardiography

The systolic ventricular function of the mice was assessed via echocardiography with a 12 MHz phased-array transducer (Philips Envisor Amsterdam, Netherlands), following a previously described method [32]. For anesthesia, the mice received 5% isoflurane during the induction phase, which was subsequently reduced to 1–1.5% for maintenance throughout the procedure. The chest area was depilated with a chemical hair remover, and ultrasound gel was applied to improve ventricular visualization. Short-axis M-mode images showing both papillary muscles were used to measure left ventricular internal diameter at diastole and systole (LVIDd and LVIDs) and LV posterior wall end-diastolic thickness (LVPWTd). Fractional shortening (%FS) was determined using the formula: %FS = [(LVIDd − LVIDs)/LVIDd] × 100. Ejection fraction (EF) was calculated as [(LV end-diastolic volume (LVEDV) − LV end-systolic volume (LVESV))/LVEDV] × 100 (%). An individual blinded to the experimental mouse groups conducted the measurements.

### Histology

Histological examination was performed as previously described [33]. Hearts were excised and transversely sectioned at the level of the papillary muscles. Specimens underwent fixation in 10% formalin and paraffin embedding. Hematoxylin and eosin (HE) and Masson trichrome (MT) staining procedures were conducted. Measurements of myocardial cell diameter cross-sections and perivascular fibrotic regions were performed following established methods [9].

The cardiomyocytes were immunostained with mouse anti-α-actinin antibody (Sigma-Aldrich) and Alexa Fluor 555-conjugated anti-mouse IgG (Thermo Fisher Scientific). PRMT5 was immunostained with a rabbit anti-PRMT5 antibody (GeneTex Inc., Irvine, CA, USA) and Alexa Fluor 647-conjugated anti-rabbit IgG (Thermo Fisher Scientific). The cells were observed under a BZ-X810 microscope (KEYENCE, Tokyo, Japan). The surface area of 20 individual myocytes was measured using ImageJ software.

### RT-qPCR

RT-qPCR analysis was performed with slight modifications to the previously described protocol [34]. Briefly, total RNA was purified using TRI Reagent (Sigma-Aldrich), and reverse transcription was subsequently carried out using ReverTra Ace qPCR RT Master Mix (TOYOBO, Osaka, Japan). For real-time PCR analysis, a LightCycler^®^ 96 Real-Time PCR System (Roche, Tokyo, Japan) was used in conjunction with KOD SYBR qPCR Mix (TOYOBO). The primer sequences employed in this study are listed in Supplementary Table 1. Relative quantification of the results was conducted using the comparative Ct method.

### Immunoprecipitation and western blot

Cells were rinsed with ice-cold PBS, and nuclear extracts were obtained from HEK293T cells as described previously [8]. A BCA protein assay kit (PIERCE, Tokyo, Japan) was utilized to assess the protein concentration of the extracted protein samples. Anti-HA-tag pAb-Agarose (MBL Life Science, Tokyo, Japan) and anti-FLAG M2 affinity gel (Cat #A2220, Sigma-Aldrich) were used for immunoprecipitation. Normal immunoglobulin served as a negative control (MBL Life Science). Nuclear extracts were added to appropriate antibodies and incubated with gentle rotation for 2 h. After the agarose beads were washed four times, the immunoprecipitants were eluted by boiling for 5 min and subjected to Western blotting analysis. Histones extracted from the cultured cells and mouse hearts were prepared as described previously [13]. Western blotting was carried out with some modifications to the previously described method [35]. Images were captured using an Amersham Imager 680 (Cytiva, Tokyo, Japan). For Western blotting analysis, the primary antibodies used in this study were an anti-DDDDK-tag mAb (Cat#M185-3L, MBL Life Science), anti-HA-tag mAb (Cat#M132-3, MBL Life Science), anti-Myc-tag mAb (Cat#M192-3, MBL Life Science), anti-PRMT5 antibody (Cat#07-405, Merck, Tokyo, Japan), Symmetric Di-Methyl Arginine Motif Rabbit mAb (#13222, Cell Signaling Technology, Tokyo, Japan), anti-β-actin mouse monoclonal clone AC-15 IgG (Cat#A1978, Sigma-Aldrich), anti-GAPDH rabbit polyclonal antibody (10494-1-AP, Proteintech), anti-histone-H3 polyclonal antibody (17168-1-AP, Proteintech), acetyl-Histone H3 (Lys9) rabbit mAb (#9649, Cell Signaling Technology), rabbit polyclonal anti-acetyl-H3 (K14) antibody (#7627, Cell Signaling Technology), rabbit anti-dimethyl-histone H3 (Arg2) antibody (Cat#07-585, Merck), rabbit polyclonal anti-H4R3me2s antibody (Cat#AB_2793544, Active motif, Tokyo, Japan), and anti-Histone H3 (acetyl K122) antibody (ab33309, Abcam, Cambridge, UK). Horseradish peroxidase (HRP)-conjugated antibodies (MBL) were employed as secondary antibodies.

### GST pull-down assay

BL21(DE3) competent *E. coli* cells were used to prepare recombinant proteins, and a pull-down assay using the GST-tag was conducted according to the previously reported method [11]. Briefly, glutathione-Sepharose 4B beads (Cytiva) were used to immobilize GST fusion proteins. Equivalent quantities of GST alone or GST fusion proteins on glutathione-agarose beads were combined with His6 fusion proteins. The mixtures underwent gentle rotation at 4 °C for 2 h. After four washes, the binding proteins were extracted and separated by SDS-PAGE. Coomassie Brilliant Blue staining was employed to visualize GST fusion proteins. Western blotting for His-tagged proteins (#2365, Cell Signaling Technology) was performed to detect interaction among the proteins.

### In vitro methylation assay

*E. coli* transformed with pGEX expression vectors were used to generate GST-tagged p300 segments, which were then purified using glutathione Sepharose 4B (GE Healthcare). HEK293T cells were used to express Flag-tagged PRMT5, which was purified with anti-FLAG M2 agarose affinity gel. The purified PRMT5 was then incubated with GST-p300 segments in reaction buffer, along with 2 μCi [^14^C]-labeled S-adenosyl-methionine (SAM) (Perkin-Elmer, Yokohama, Japan) at 30 °C. SDS-PAGE sample buffer was used to quench the reaction mixture, which was then separated by SDS-PAGE and transferred to a polyvinylidene fluoride (PVDF) membrane (Merck). The BAS2000 system (Fujifilm, Tokyo, Japan) was used to fluorograph the membrane.

### In vitro histone acetyltransferase assay

Nuclear extracts were isolated from hearts and applied to a 96-well plate. A histone acetyltransferase assay kit was used to determine HAT activity in mouse hearts, following the manufacturer’s protocol (#56100, Active Motif).

HA-tagged p300 was expressed in HEK293T cells, purified with HA-tag pAb-Agarose (MBL Life Science), and incubated with recombinant Histone H3.1 (NEB Biolabs #M2503, Tokyo, Japan), in reaction buffer, in the presence of [^3^H]-labeled acetyl-CoA (American Radiolabeled Chemicals, Saint Louis, USA, 0.3 µCi) at 30 °C. To quench the reaction, the mixture was added to SDS-PAGE sample buffer. The histone was then separated by SDS-PAGE and transferred to a PVDF membrane. CBB staining was used to observe the histone on the membrane, and a liquid scintillation counter (LSC-7400, Hitachi-Aloka Medical, Tokyo, Japan) was employed to measure radioactivity.

### Statistical analyses

Data are expressed as means ± standard error. GraphPad Prism 9 (GraphPad Software, Inc., San Diego, USA) was utilized for statistical analysis. The Shapiro–Wilk normality test was employed to assess data normality. Significant differences were assessed using two-tailed unpaired Student’s *t*-tests or one-way analysis of variance (ANOVA) with Tukey’s *post-hoc* test or Dunnett’s *post-hoc* test. Differences were considered to be statistically significant when the p-values were less than 0.05.

## Results

### Cardiac overexpression of PRMT5 accelerates left ventricular dysfunction after pressure overload

**Fig. 1:**
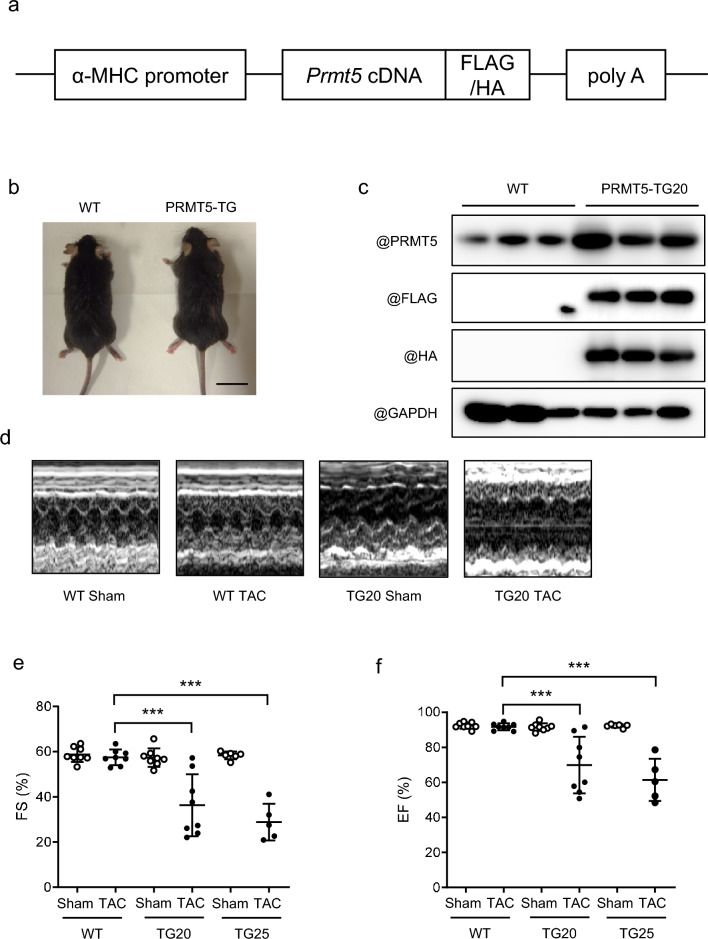
Cardiac-specific overexpression of PRMT5 accelerates pressure overload-induced cardiac systolic dysfunction. **a** A schematic diagram of the transgene to create mice with cardiac-specific *Prmt5* overexpression (PRMT5-TG). **b** Images of WT and PRMT5-TG mice littermates at 10 weeks of age. Scale bar: 20 µm. **c** Cardiac PRMT5 overexpression confirmed using Western blotting. **d** Echocardiographic analysis images of PRMT5-TG mice performed 4 weeks after TAC surgery. **e**, **f** Fractional shorting (**e**) and ejection fraction (**f**) calculated from M-mode echocardiography. Values are presented as mean ± SD (n = 6–8 mice/group). Data are analyzed using two-way ANOVA, followed by Tukey’s multiple comparison test. A *p* < 0.05 is considered statistically significant. ***p < 0.001

During myocardial hypertrophy and heart failure, various gene expressions are altered in the heart through epigenetic regulatory mechanisms, including histone post-translational modifications [3]. In our previous study, treatment with a PRMT5 inhibitor EPZ015666 suppressed pressure overload-induced cardiac hypertrophy [27], indicating that PRMT5 in cardiomyocytes may accelerate the cardiac hypertrophy and systolic dysfunction caused by pressure overload. To investigate the effects of PRMT5 gain-of-function on cardiac function in mice, we have genetically engineered transgenic mice to express PRMT5 specifically in cardiomyocytes (PRMT5-TG, Fig. 1a). These mice were born and grew similarly to their wild-type (WT) counterparts (Fig. 1b). We confirmed that the expression of PRMT5 was significantly higher in the hearts of the PRMT5-TG mice than in the hearts of the WT mice (Fig. 1c, Supplementary Figs. 1 and 2). To address whether PRMT5 overexpression contributes to pressure overload-induced cardiac systolic dysfunction, we conducted echocardiography in the WT and two lines of PRMT5-TG: TG20 and TG25. At 4 weeks post-TAC surgery, the index of systolic function (FS and EF) was significantly worse in PRMT5-TG mice as compared to the WT mice (Fig. 1d, e, and Supplementary Table 2). These data suggest that cardiac-specific PRMT5 overexpression aggravates pressure overload-induced cardiac dysfunction.

### Cardiac overexpression of PRMT5 promotes myocardial hypertrophy induced by pressure overload

**Fig. 2:**
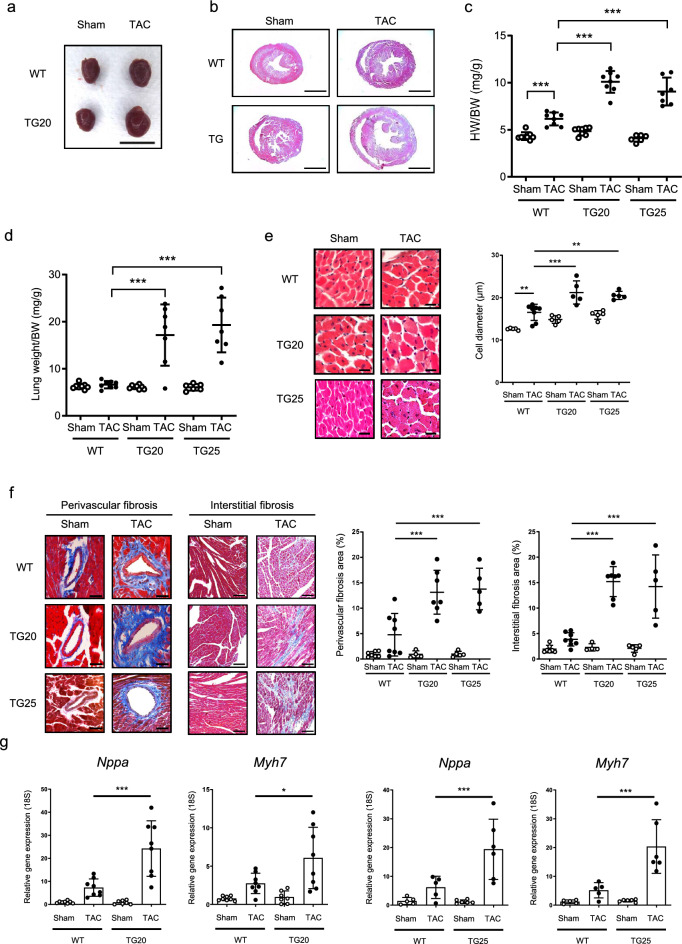
Cardiac overexpression of PRMT5 promotes pressure overload-induced cardiac hypertrophy. **a** Representative photos of the hearts extracted from the mice. **b** Histological analysis of the heart tissues. Scale bars: 2 mm. **c** Heart weight/body weight comparisons at 4 weeks after TAC surgery. Values are presented as mean ± SD (n = 6–8 mice/group) **d** Cardiac overexpression of PRMT5 promotes the increases in lung weight after TAC surgery. **e**, **f** Histological analysis results of PRMT5-TG mice at 4 weeks after TAC surgery. Representative images of HE-stained sections of the mice hearts. Cross-sectional myocardial cell diameter measurement (**e**). Representative images of MT-stained perivascular and interstitial fibrosis area of the LV myocardium of the mice. Perivascular and interstitial fibrotic area measurements (**f**). Values are presented as mean ± SD (n = 5 mice/group). Scale bars: 20 µm (**e**), 50 µm (Perivascular) and 100 µm (interstitial) (**f**). **g** Hypertrophic gene expression is increased by cardiac overexpression of PRMT5. Values are expressed as mean ± SD (n = 7–8 mice/group). Data are analyzed using two-way ANOVA, followed by Tukey’s multiple comparison test. A *p* < 0.05 is considered statistically significant. *p < 0.05, **p < 0.01, *** *p* < 0.001

Cardiac hypertrophy is a critical indicator of pressure overload-induced contractile dysfunction. Since the overexpression of PRMT5 in cardiac myocytes accelerated cardiac systolic dysfunction, we assessed cardiac hypertrophy in mice. In WT mice, the heart weight to body weight (HW/BW) ratio was substantially elevated by TAC surgery. Although the HW/BW ratio was not considerably different between WT and PRMT5-TG mice under baseline conditions, pressure overload-induced cardiac hypertrophy was markedly exacerbated in the PRMT5-TG mice as compared to the WT mice (Fig. 2a–c). Pulmonary edema-associated congestive heart failure led to an increase in lung weight. Consistent with the results that cardiac function was reduced by PRMT5 overexpression, the lung weight to body weight ratio was significantly augmented (Fig. 2d), suggesting that PRMT5-TG mice had accelerated heart failure compared with WT mice. Histological analyses showed that cardiac cell diameter and perivascular and interstitial fibrotic area were significantly increased in PRMT5-TG mice with pressure overload (Fig. 2e, f). We examined atrial natriuretic peptide (ANP) and β-MHC gene expression in mouse hearts using RT-qPCR. These gene transcript expression levels were increased in response to pressure overload, and these increases were significantly promoted by the cardiac overexpression of PRMT5 (Fig. 2g). These data indicate that PRMT5 overexpression in cardiac myocytes exacerbates pathological cardiac hypertrophy in vivo.

### PRMT5 loss of function suppresses cardiomyocyte hypertrophy in vitro

**Fig. 3:**
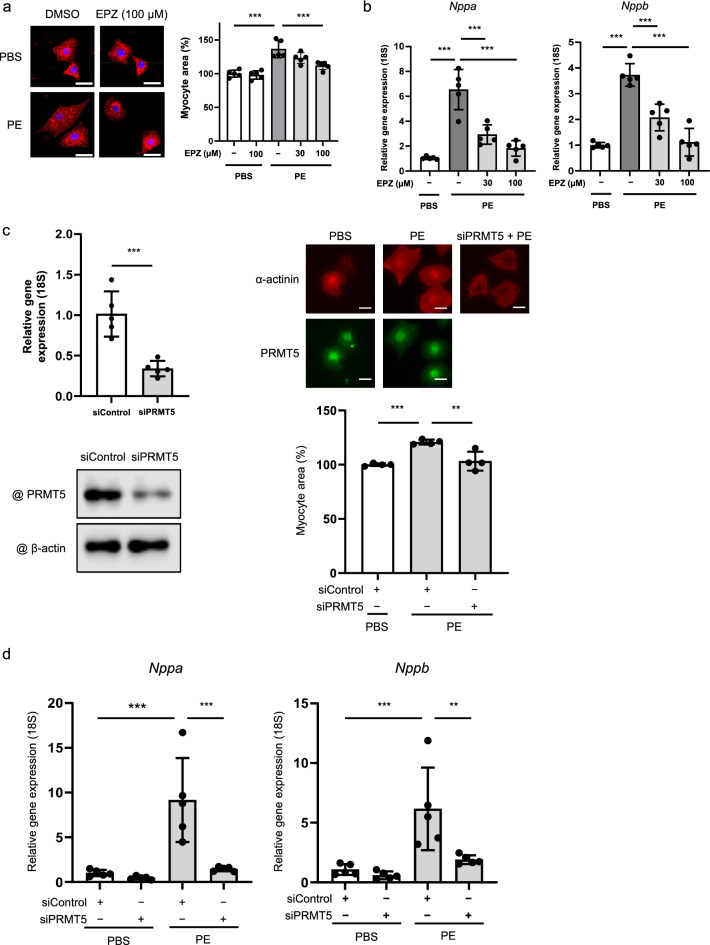
Pharmacological inhibition and knockdown of PRMT5 suppresses phenylephrine (PE)-induced hypertrophic responses in cultured cardiomyocytes. **a** Immunostaining images for α-actinin to determine the cell surface area of cardiomyocytes, quantified using ImageJ software. Scale bars: 20 µm. **b** Hypertrophic gene expression levels of *Nppa* and *Nppb*, quantified by qRT-PCR. **c** Primary cultured cardiomyocytes were transfected with siRNA (siControl or siPrmt5) and then stimulated with or without PE (30 µM). *Prmt5* knockdown is confirmed by qRT-PCR and WB. The cell surface area was quantified using ImageJ software. Scale bars: 20 µm. **d** PE-induced *Nppa* and *Nppb* gene expression as quantified by qRT-PCR and Western blotting. Values are presented as mean ± SD (n = 4–5). Data are analyzed using one-way ANOVA, followed by Dunnett’s multiple comparison tests versus the PE-treated group. *p < 0.05, **p < 0.01, ***p < 0.001

PRMT5 overexpression in hearts promoted pressure overload-induced myocardial hypertrophy. These experimental findings suggest that PRMT5 gain-of-function in cardiomyocytes accelerates cardiomyocyte hypertrophy. Therefore, we utilized primary cultured cardiomyocytes to explore how the loss of PRMT5 function affects cell hypertrophy. The cardiomyocytes from neonatal rats were hypertrophied by phenylephrine (PE), and this cardiac cell hypertrophy was significantly prevented by treatment with the specific inhibitor of PRMT5: EPZ015666 (Fig. 3a). During cardiomyocyte hypertrophy, the expression of various fetal genes is induced. PE treatment induced the expression of ANP and B-type natriuretic peptide, which was significantly suppressed by EPZ015666 treatment (Fig. 3b). Similarly, PRMT5 knockdown attenuated myocardial cell hypertrophy and hypertrophic gene transcription in response to PE treatment (Fig. 3c, d), suggesting that PRMT5 loss-of-function mitigates pathological cardiomyocyte hypertrophy.

### Cardiac overexpression of PRMT5 increases histone H3K9 acetylation

**Fig. 4:**
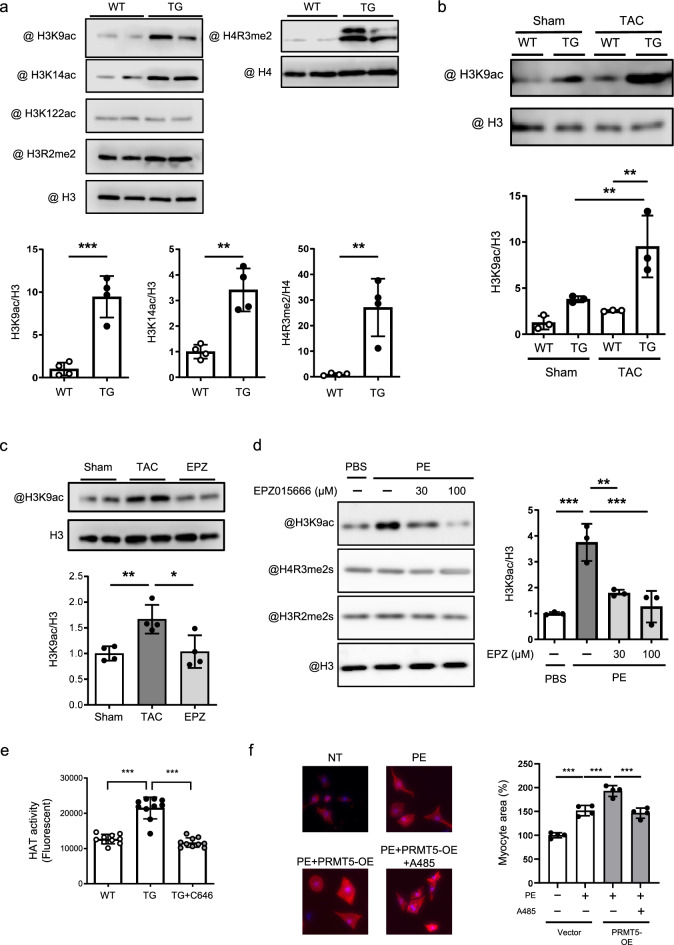
PRMT5 modifies histone methylation and acetylation in hearts and cultured cardiomyocytes. **a** Acid extracts from mouse hearts of WT and PRMT5 transgenic (PRMT5-TG) mice were applied to Western blotting analysis. Western blotting was performed using the indicated antibodies. **b** WT and PRMT5-TG mice were subjected to the TAC surgery. The samples prepared from these hearts were used for Western blotting. **c** Western blotting was performed using acid extracts from mouse hearts of sham and TAC mice treated with or without the PRMT5 inhibitor EPZ015666. Values are presented as mean ± SD (n = 4 mice). **d** Cultured cardiomyocytes were treated with EPZ015666 in the presence or absence of PE. Values are presented as mean ± SD (n = 3). **e** HAT activity in hearts was measured using a fluorescent-based method. A p300 HAT inhibitor, C646, was added to the protein extracts from the hearts of PRMT5-TG mice. Values are presented as mean ± SD (n = 10 mice/group). **f** Immunostaining images for α-actinin to determine the cell surface area of cardiomyocytes, quantified using ImageJ software. Scale bars: 20 µm. Values are presented as mean ± SD (n = 4). Data are analyzed using one-way ANOVA, followed by Tukey’s multiple comparison (**c**, **e**, **f**) or Dunnett’s multiple comparison tests versus the PE-treated group (**d**). *p < 0.05, **p < 0.01, ***p < 0.001

Since PRMT5 gain-of-function accelerates cardiomyocyte hypertrophy, we investigated the molecular mechanism through which PRMT5 promotes hypertrophic gene transcription. Gene transcription is linked to histone modifications such as acetylation and methylation. We examined histone arginine methylation and lysine acetylation using Western blotting. The results showed that H3K9 and H3K14 acetylation and H4R3 arginine dimethylation were significantly enhanced in the hearts of PRMT5-TG mice (Fig. 4a). Previous studies have identified p300-mediated acetylation of the histone H3K9 residue as an important histone modification in cardiac hypertrophy and hypertrophic gene transcription [13]. Consistent with previous studies, pressure overload-induced H3K9 acetylation was enhanced by cardiac overexpression of PRMT5 (Fig. 4b). In addition, the PRMT5 inhibitor EPZ015666 suppressed H3K9 acetylation induced by both pressure overload and PE (Fig. 4c, d). HAT activity in the nuclear fraction of the heart was determined. HAT activity was significantly elevated in PRMT5-TG hearts, and the increase in HAT activation resulting from PRMT5 overexpression was abolished by the p300-specific HAT inhibitor C646 (Fig. 4e). The transduction of PRMT5 using the lentivirus system promoted PE-induced cardiomyocyte hypertrophy (Supplementary Figs. 3 and 4). However, treatment with a cell-permeable p300 HAT inhibitor A485 attenuated cell hypertrophy (Fig. 4f). These data support the notion that PRMT5 overexpression promotes p300 HAT activity in the heart.

### PRMT5 methylates p300 at R200 and promotes HAT activity of p300

**Fig. 5:**
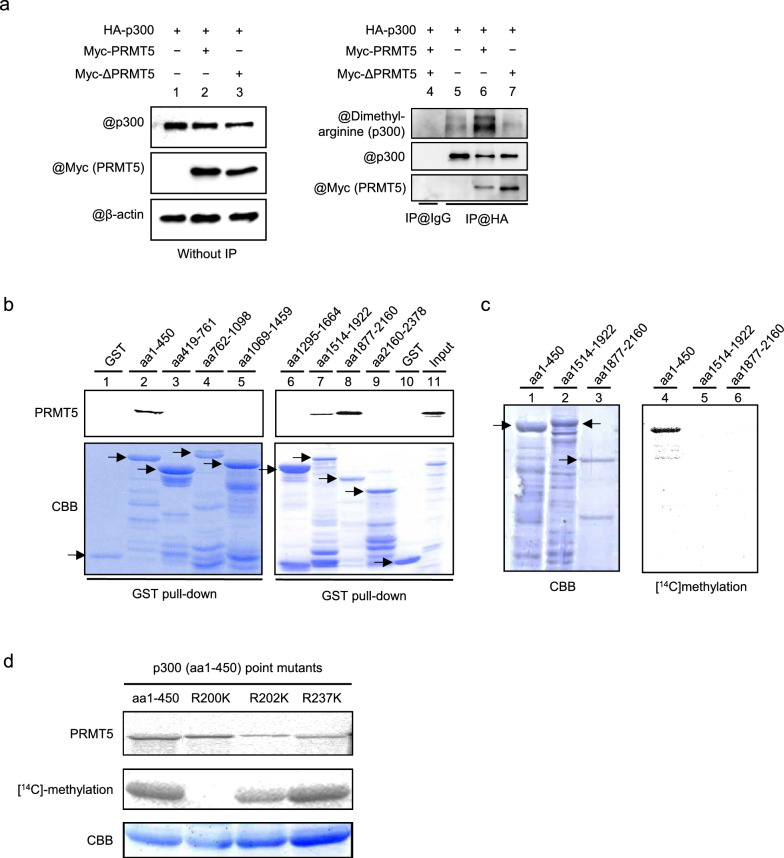
PRMT5 methylates p300 at R200. **a** The plasmids of WT PRMT5 and the deletion mutant lacking enzymatic activity of PRMT5 (ΔPRMT5) were transfected into HEK293T cells. Immunoprecipitation of p300 and Western blotting was performed. **b** GST pull-down assay was performed with GST-fusioned aa1-450, aa1514-1922, and aa1817-2160 of p300 mutants extracted from *E. coli*. [^35^S]radiolabeled PRMT5 was obtained using an in vitro translation system. The arrows show GST fusion proteins. **c** PRMT5 and aa1-450, aa1514-1922, and aa1817-2160 of p300 mutants were mixed in reaction solution with [^14^C]radiolabeled S-adenosyl methionine (SAM). The proteins were analyzed using SDS-PAGE and scanned using BAS2000. **d** PRMT5 and R200K, R202K, and R237K of p300 aa1-450 point mutants were mixed in reaction solution with [^14^C]radiolabeled SAM, and an in vitro methylation assay was performed

PRMT5 binds and methylates histones and some functional proteins and regulates biological processes [36]. The interaction between p300 and PRMT5 and the p300 symmetric arginine dimethylation via PRMT5 were examined in HEK293T cells. Full-length PRMT5 and a methyltransferase-deficient PRMT5 mutant were overexpressed in HEK293T cells to examine their interaction with and methylation of p300. PRMT5 and PRMT5 mutant interacted with p300. PRMT5 methylated p300 but the PRMT5 mutant did not (Fig. 5a). To determine whether PRMT5 binds and methylates p300, GST pull-down and in vitro methylation assays were carried out. GST pull-down assay showed that PRMT5 bound to the p300 aa1-450, aa1514-1922, and aa1877-2180 fragments (Fig. 5b). Among them, we found that the fragment aa1-450 was methylated by PRMT5 in vitro (Fig. 5c). A previous report showed that CBP-1 at R234 was methylated by PRMT5 in *Caenorhabditis elegans* [37] and that the corresponding amino acid residue is within a GRG sequence that is also found at the N-terminal region of human p300 proteins. To determine which arginine residue is methylated by PRMT5, we generated the point mutation mutants of p300 and examined them using an in vitro methylation assay. Substitution of arginine 200 with lysine (R200K) completely abolished the ability of PRMT5 to methylate the N-terminus of p300 (Fig. 5d), indicating that the R200 residue is essential for the arginine methylation of p300, which is mediated by PRMT5. These data imply that PRMT5 associates with p300 and symmetrically dimethylates the R200 of p300.

**Fig. 6:**
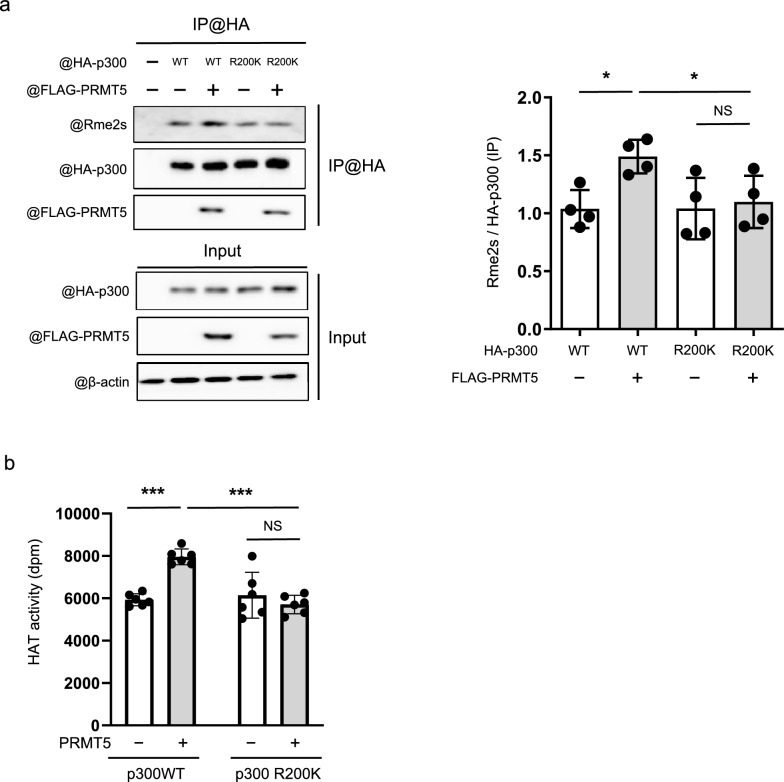
PRMT5-mediated p300 arginine methylation of R200 is required for p300 histone acetyltransferase activity. **a** The plasmids encoding p300 WT or p300 R200K point mutant and PRMT5 were transfected to HEK293T cells. Immunoprecipitation by anti-HA antibody followed by Western blotting was performed. The band density was measured using ImageJ software. Values are presented as mean ± SD (n = 3). **b** p300 WT and p300 R200K point mutant were purified using anti-HA-tag agarose beads from HEK293T cells co-transfected with or without PRMT5. Purified p300 was incubated with recombinant histone H3 peptide and [^3^H]-labeled acetyl-CoA. The histone H3 peptides were isolated, and radioactivity was measured using a liquid scintillation counter. Values are presented as mean ± SD (n = 6). Data are analyzed using two-way ANOVA, followed by Tukey’s multiple comparison test. *p* < 0.05 was considered statistically significant. *p < 0.05, ***p < 0.001

We focused on the histone acetyltransferase activity of p300, which activates gene transcription by acetylating H3K9. We examined whether the R200 point mutant of p300 abolished PRMT5-mediated methylation of p300. PRMT5, p300 WT, and R200K mutant were overexpressed in HEK293T cells. Although PRMT5 enhanced the methylation of the full-length p300, the R200K mutation in p300 failed to improve methylation by PRMT5 (Fig. 6a). We then tested whether PRMT5 activates HAT in the wild-type p300 and in the p300 R200K mutant. The co-expression of p300 WT and PRMT5 enhanced HAT activity, whereas R200K mutant did not (Fig. 6b). Collectively, these findings indicate that R200 in p300 is the critical arginine residue methylated by PRMT5 and that this methylation is required for PRMT5-mediated enhancement of p300 HAT activity.

## Discussion

**Fig. 7:**
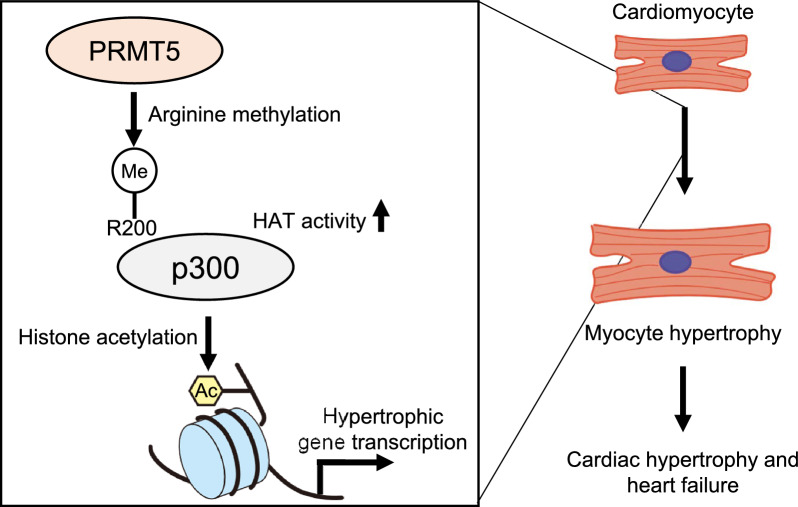
Graphical abstract. The gain-of-function of PRMT5 accelerates pressure overload-induced cardiomyocyte hypertrophy and heart failure. PRMT5 methylates p300 at R200 and regulates p300 HAT activity, which is essential for histone acetylation during the development of cardiomyocyte hypertrophy

Epigenetic modifications have been implicated in pathological cardiac remodeling [7], suggesting that epigenetic regulators could serve as therapeutic molecular targets for chronic heart failure. Among epigenetic modifiers, we have previously demonstrated that p300 is essential in post-myocardial infarction remodeling, and the pharmacological inhibition of p300 using curcumin suppresses myocardial infarction-induced LV remodeling and dysfunction. Although it has been reported that lysine methyltransferases are also crucial in these processes, the precise roles of arginine methylation on cardiac function are unclear. In this study, we demonstrated that (1) overexpression of PRMT5 in cardiomyocytes accelerates cardiac hypertrophy and LV dysfunction in mice undergoing TAC surgery, (2) EPZ015666, a selective PRMT5 inhibitor, or PRMT5 knockdown abrogates PE-induced cardiomyocyte hypertrophy, and (3) PRMT5 symmetrically methylates p300 at R200, enhancing its HAT activity and promoting H3K9 acetylation. Our experimental findings suggest that the PRMT5/p300 epigenetic protein complex regulates pathological cardiac remodeling and chronic systolic dysfunction (Fig. 7).

PRMT5 cardiac-specific overexpression resulted in enhanced acetylation of H3K9, a target residue of p300 [13], and treatment with a PRMT5-specific inhibitor reduced it. HAT activity was elevated in PRMT5-TG hearts compared to WT hearts, and this increase was abrogated by the addition of the p300 inhibitor C646. These data imply that PRMT5 increases p300 HAT activity in cardiomyocytes. In a previous study, cardiac-specific p300 transgenic mice did not exhibit cardiac remodeling at least at the age of 17 weeks under physiological conditions but did promote myocardiac infarction-induced heart failure [38]. The data suggest that p300 would be a mediator of pathological cardiac hypertrophy, and another signal activation stimulus is necessary for induction in addition to p300 activation. We, and other groups, have shown that p300 HAT activity is needed to cause pathological cardiac remodeling and dysfunction through the acetylation of histones and a transcription factor GATA-4 [9, 38]. It has been reported that post-transcriptional modifications, including phosphorylation, methylation, and acetylation, can regulate HAT activity and p300 function [39–41]. In *Caenorhabditis elegans*, PRMT5 methylates CBP-1—the nematode ortholog of p300/CBP in humans—and regulates p53-dependent apoptosis [37]. CBP/p300 is asymmetrically methylated by arginine methyltransferase 4 (PRMT4/CARM1), which modulates its HAT activity and gene transcription [39, 42]. Moreover, CARM1-mediated methylation of p300 has been shown to hinder its interaction with glucocorticoid receptor-interacting protein, thereby regulating transcription [43]. The inhibition of PRMT1, an enzyme that asymmetrically methylates arginine residues, also represses c-myc-dependent transcription by decreasing its interaction with p300 [44]. Our results showed that PRMT5 symmetrically methylated p300 at R200 and increased HAT activity. As R200 is not located within the HAT domain of p300, its arginine methylation may not directly influence p300 HAT activity [45]. The N-terminus of p300 contains a nuclear-receptor-interaction domain [45]. This domain is known to interact with the estrogen receptor, thyroid hormone receptor, retinoic X receptor, and retinoic acid receptor and to control their transcriptional activity [46]. It is possible that PRMT5-mediated arginine methylation of p300 alters the protein–protein interactions involved in the p300 HAT activity. In our study, PRMT5 overexpression led to histone acetylation under physiological conditions. However, the pathophysiological role of PRMT5-mediated p300 methylation in cardiomyocytes has not yet been fully elucidated.

Can PRMT5 directly acetylate histone tails in cardiomyocytes? To our knowledge, no study has reported that PRMT5 can acetylate histones or other proteins. Although our findings suggest that p300, which acetylates histone tails including H3K9 and H3K14, is a key mediator of PRMT5-induced cardiac remodeling, studies have shown that crosstalk between histone methylation and acetylation underlies numerous biological and pathological processes [16, 47]. As H3K9 and H3K14 typically co-regulate gene transcription, it is possible that H3K14 acetylation is caused by p300 activation and promotes hypertrophic gene transcription. Our results showed that symmetric H4R3 dimethylation, a repressive histone mark mediated by PRMT5 [36], was markedly increased; whereas H3R2me2s, which enhances gene transcription [48], remained unchanged in PRMT5-overexpressing cardiomyocytes. H4R3me2 is a histone mark known to repress gene transcription. Although the functions in cardiac hypertrophy are still unknown, H4R3me2 does not seem to contribute to our conclusion that PRMT5 promotes hypertrophic gene transcription. Although further investigation is required to determine whether crosstalk between H4R3 di-methylation and histone acetylation affects heart failure development, our data suggest that histone methylation by PRMT5 may not be the primary driver of PRMT5-induced hypertrophy.

The functions of epigenetic writers, readers, and erasers in heart diseases have been studied, and epigenetic regulators have been identified as potential therapeutic targets in heart failure [10, 49]. Our previous studies have revealed that the pharmacological abrogation of p300 HAT activity using curcumin attenuates cardiac hypertrophy and systolic dysfunction induced by myocardial infarction [9]. Class I histone deacetylase inhibitors present a beneficial effect on cardiac hypertrophy and left ventricular dysfunction in pathological conditions [7, 50]. Several investigations have highlighted the potential of the bromodomain and extraterminal (BET) inhibitor JQ1 in mitigating pathological cardiac remodeling [51, 52]. The histone methylation is a key factor in the development of congestive heart failure. A histone methyltransferase G9a, known to mono- and di-methylate H3K9, is required to promote pathological cardiac hypertrophy, and the treatment with its specific inhibitor BIX-01294 has improved cardiac dysfunction in response to pressure overload [53]. In addition to these therapeutic candidates, our findings in this study propose the potential of PRMT5 as a novel therapeutic target in heart failure. Recently developed PRMT5 inhibitors—some already investigated in clinical studies for the treatment of solid tumors—appear to have manageable toxicity profiles [54, 55]. However, since these epigenetic regulators, including PRMT5, also function under normal physiological conditions, potential adverse effects are a concern for chronic diseases. Further studies are needed to analyze the undesired effects in both animal and human studies carefully and to investigate a molecular-specific implication in the pathogenesis of heart diseases.

Here, we have demonstrated that PRMT5 overexpression in cardiomyocytes promotes pressure overload-induced myocardial hypertrophy. In this study, our experimental data suggest that PRMT5 in cardiomyocytes contributes to pathological cardiac hypertrophy through epigenetic activation of hypertrophic gene transcription. Previous studies have also shown the role of PRMT5 in cardiac myocytes. Cardiac-specific *Prmt5* knockout results in a pathological condition of dilated cardiomyopathy without induction of pathological conditions in adult mice [26]. This suggests that PRMT5 serves an essential role in maintaining the physiological cardiac function and that PRMT5 selective inhibitors may induce cardiac toxicity. This study also reported that cardiomyocyte-specific knockout of PRMT5 suppressed isoproterenol-induced cardiomyocyte hypertrophy, which is consistent with our conclusion. Chen et al. reported that PRMT5 methylates GATA4, a transcription factor involved in hypertrophic gene expression, and decreases its transcriptional activity [25]. Cai et al. reported that the symmetric dimethylation of histone H4R3 caused by PRMT5 may be necessary in regulating pathological left ventricular hypertrophy [24]. These results are inconsistent with those of this study. Further studies are needed to confirm the function of PRMT5 in cardiomyocytes. PRMT5 has many interaction partners and protein substrates [56, 57], indicating that PRMT5 regulates various signaling pathways under specific conditions. Consequently, PRMT5 likely participates in multiple signaling pathways in cardiomyocytes, playing distinct roles in physiological versus pathological states.

## Conclusion

The gain-of-function of PRMT5 in cardiomyocytes accelerates pressure overload-induced cardiac hypertrophy and left ventricular systolic dysfunction through the regulation of p300 HAT activity. Our findings suggest that symmetric arginine methylation of p300 plays a crucial role in cardiac hypertrophy. Our experimental findings also propose that PRMT5 is a novel molecular target for the pharmacological therapy of chronic heart failure. However, as PRMT5 is essential for cardiac maintenance [26], inhibiting it may lead to adverse effects. Further long-term trials are required to investigate the cardiac toxicity of PRMT5 inhibitors. Growing evidence on the potential involvement of epigenetic enzymes in heart diseases may aid in the development of novel clinical tools.

## Supplementary Information

Additional file 1.

Additional file 2.

## Data Availability

This published article and its supplementary information files include all data generated or analyzed during this study.

## Abbreviations

ANP: Atrial natriuretic peptide
β-MHC: β-Myosin heavy chain genes
FS: Fractional shortening
EF: Ejection fraction
HAT: Histone acetyltransferase
LVPWTd: Left-ventricular posterior wall thickness diameter
LVEDD: Left-ventricular end-diastolic dimensions
LVESD: Left-ventricular end-systolic internal dimensions
ANOVA: One-way analysis of variance
PRMT5: Protein arginine methyltransferase 5
PRMT5-TG: Transgenic (mice)
SAM: S-adenosyl-methionine
TAC: Transverse aortic constriction
