# Correction: Cardiac-specific overexpression of PRMT5 exacerbates pressure overload-induced hypertrophy and heart failure

**DOI:** 10.1186/s12929-025-01174-2

**Published:** 2025-08-26

**Authors:** Yasufumi Katanasaka, Yoichi Sunagawa, Ryoga Sakurai, Mikuto Tojima, Ryuya Naruta, Yuya Hojo, Yuto Kawase, Toshihide Hamabe‑Horiike, Kiyoshi Mori, Koji Hasegawa, Tatsuya Morimoto

**Affiliations:** 1 Division of Molecular Medicine, School of Pharmaceutical Sciences, University of Shizuoka, 52‑1 Yada, Suruga‑ku, Shizuoka, 422‑8526 Japan; 2 Division of Translational Research, National Hospital Organization Kyoto Medical Center, 1‑1 Mukaihata‑cho Fukakusa, Fushimi‑ku, Kyoto, 612‑8555 Japan; 3 Shizuoka General Hospital, 4‑27‑1 Kita Ando Aoi‑ku, Shizuoka, 420‑8527 Japan; 4 Graduate School of Public Health, Shizuoka Graduate University of Public Health, Shizuoka, 4200881 Japan; 5 Department of Molecular and Clinical Pharmacology, School of Pharmaceutical Sciences, University of Shizuoka, Shizuoka, 4228526 Japan

**Correction: Journal of Biomedical Science (2025) 32:61** 10.1186/s12929-025-01162-6

Following publication of the original article [1], an error was identified in Fig. 1. The correct Fig. 1 is shown below. Besides, another Additional File 2 which included a dataset supporting the conclusions is added.

**Fig. 1:**
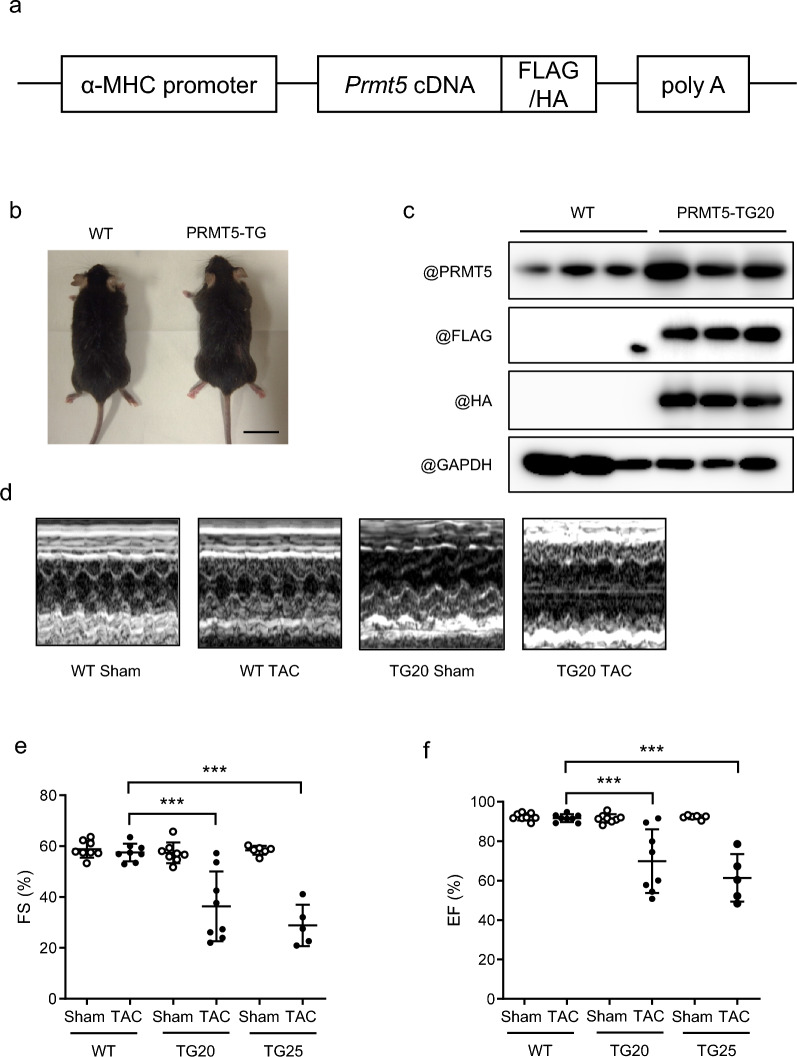
Cardiac-specific overexpression of PRMT5 accelerates pressure overload-induced cardiac systolic dysfunction. **a** A schematic diagram of the transgene to create mice with cardiac-specific *Prmt5* overexpression (PRMT5-TG). **b** Images of WT and PRMT5-TG mice littermates at 10 weeks of age. Scale bar: 20 µm. **c** Cardiac PRMT5 overexpression confirmed using Western blotting. **d** Echocardiographic analysis images of PRMT5-TG mice performed 4 weeks after TAC surgery. **e**, **f** Fractional shorting (**e**) and ejection fraction (**f**) calculated from M-mode echocardiography. Values are presented as mean ± SD (n = 6–8 mice/group). Data are analyzed using two-way ANOVA, followed by Tukey’s multiple comparison test. A *p* < 0.05 is considered statistically significant. ***p < 0.001

The original paper has been updated.

## Supplementary Information

Additional file 2.
