# Supplementary material for: Cardiac-specific overexpression of PRMT5 exacerbates pressure overload-induced hypertrophy and heart failure
Source: J Biomed Sci. 2025 Jul 6;32:61. doi: 10.1186/s12929-025-01162-6 (PMC12229037; doi:10.1186/s12929-025-01162-6)
Supplement: Supplementary file 1 — Additional file 1. [file 12929_2025_1162_MOESM1_ESM.pdf]

Supplementary Table 1
Primers used in this study

Name	Forward primer (5' to 3')	Reverse primer (5' to 3')
mouse-Nppa	ATTGACAGGATTGGAGCCCAGAGT	TGACACACCACAAGGGCTTAGGAT
mouse-Myh7	CCTCACATCTTCTCCATCTCTG	TTGGATGACCCTCTTAGTGTTG
rat-Nppa	AGGCCATATTGGAGCAAATC	CATCTTCTCCTCCAGGTGGT
rat-Nppb	GATTCTGCTCCTGCTTTTCC	CATCGTGGATTGTTCTGGAG
18S rRNA	CTTAGAGGGACAAGGGCG	GGACATCTAAGGGCATCACA

Supplementary Table 2
Echocardiography analysis of PRMT5 transgenic mice after TAC surgery

	Sham			TAC		
	WT	PRMT5-TG20	PRMT5-TG25	WT	PRMT5-TG20	PRMT5-TG25
IVSd, mm	0.9 ± 0.1	1.0 ± 0.2	1.0 ± 0.2	1.2 ± 0.2	1.1 ± 0.2	1.1 ± 0.1
IVSs, mm	1.6 ± 0.1	1.7 ± 0.2	1.7 ± 0.1	1.9 ± 0.4	1.6 ± 0.4	1.5 ± 0.3
LVIDd, mm	2.9 ± 0.3	3.1 ± 0.4	2.9 ± 0.2	2.8 ± 0.4	3.7 ± 0.7 #	4.0 ± 1.1 ##
LVIDs, mm	1.2 ± 0.2	1.3 ± 0.2	1.2 ± 0.1	1.2 ± 0.2	2.5 ± 1.0 ###	2.9 ± 1.1 ###
LVPWd, mm	1.1 ± 0.2	1.1 ± 0.2	1.2 ± 0.1	1.4 ± 0.1 *	1.6 ± 0.2 #	1.6 ± 0.3 #
LVPWs, mm	1.7 ± 0.3	1.8 ± 0.2	1.6 ± 0.1	2.0 ± 0.4	1.8 ± 0.6	1.7 ± 0.6
FS, %	58.8 ± 3.3	57.4 ± 4.1	58.4 ± 1.7	57.5 ± 3.5	36.3 ± 13.7 ###	28.8 ± 8.1 ###
EF, %	92.4 ± 1.8	92 ± 2.2	92.3 ± 1.0	91.7 ± 2.0	69.8 ± 16.1 ###	61.4 ± 12.1 ###

Data show the mean ± SD.
Two-way ANOVA, followed by Holm-Sidak's comparisons test.
Significant differences are indicated as follows:
* *p* <0.05 vs Sham-operated *WT* group
# *p* < 0.05, ## *p* < 0.01, ### *p* < 0.001 vs TAC-operated WT group

IVSd, interventricular septum thickness at diastolic; IVSs, interventricular septum thickness at systolic; LVIDd, left ventricular internal dimension at diastolic; LVIDs, left ventricular internal dimension at systolic; LVPWd, left ventricular posterior wall thickness at diastolic; LVPWs, left ventricular posterior wall thickness at systolic; FS, fractional shortening; EF, ejection fraction

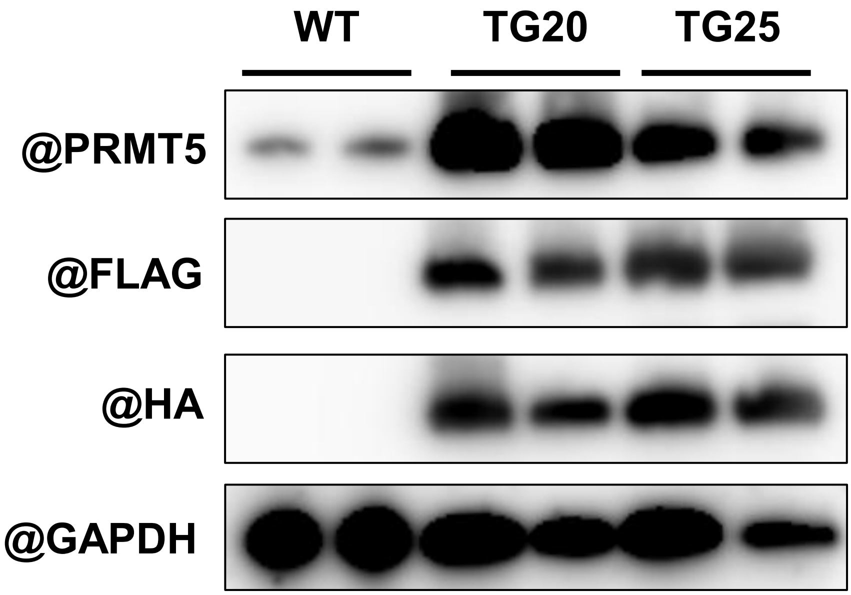

Supplementary figure 1
Exogeneous Prmt5 expression was validated using Western blot analysis. Protein extracts from the heart tissues of WT, TG20, and TG25 were applied to the analysis.

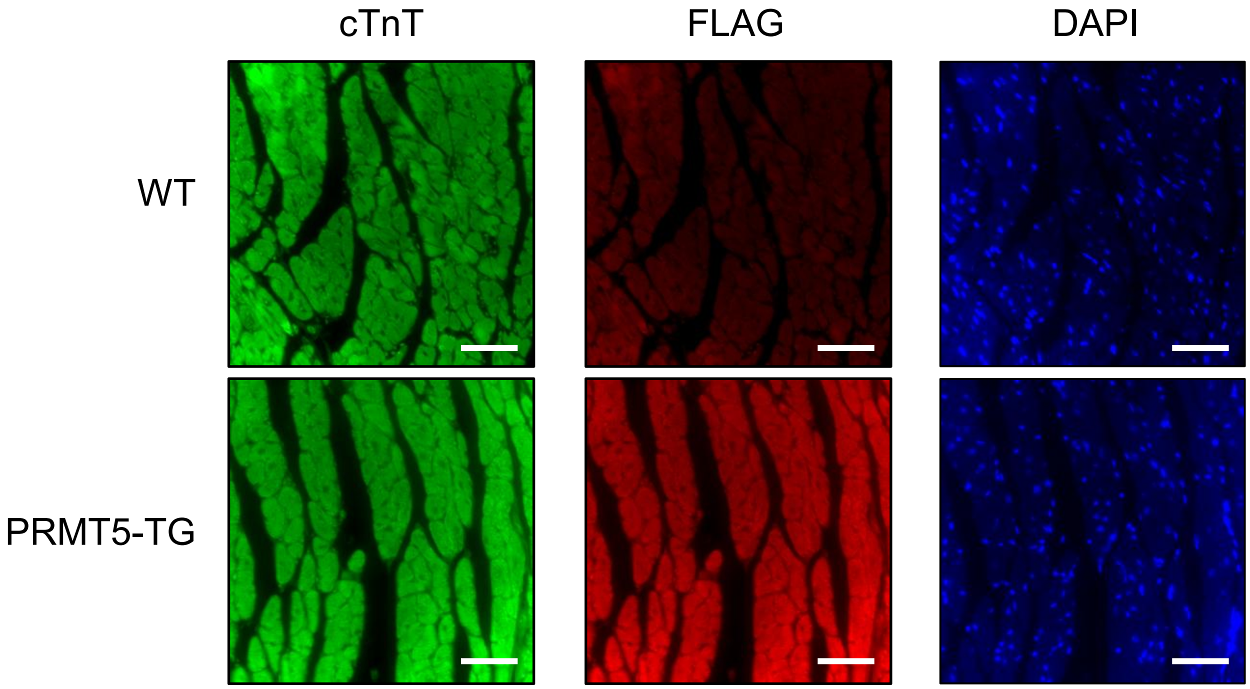

Supplementary figure 2
Exogeneous Prmt5 expression was validated using Immunofluorescent staining. Representative images of cardiomyocytes (green, Cardiac troponin T, cTnT), FLAG (red), and nucleus (blue) in heart tissues are shown. Scale bars indicate 50 μm.

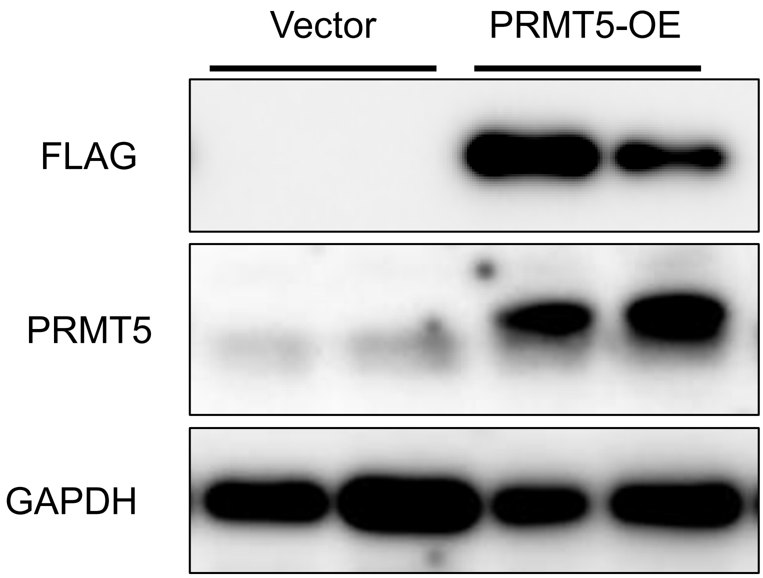

Supplementary figure 3
Exogeneous FLAG-PRMT5 expression was validated using Western blot analysis. Lentiviral transduction of FLAG-PRMT5 or vector control was performed to rat cardiomyocytes. Protein extracts from the cardiomyocytes were applied to the analysis.

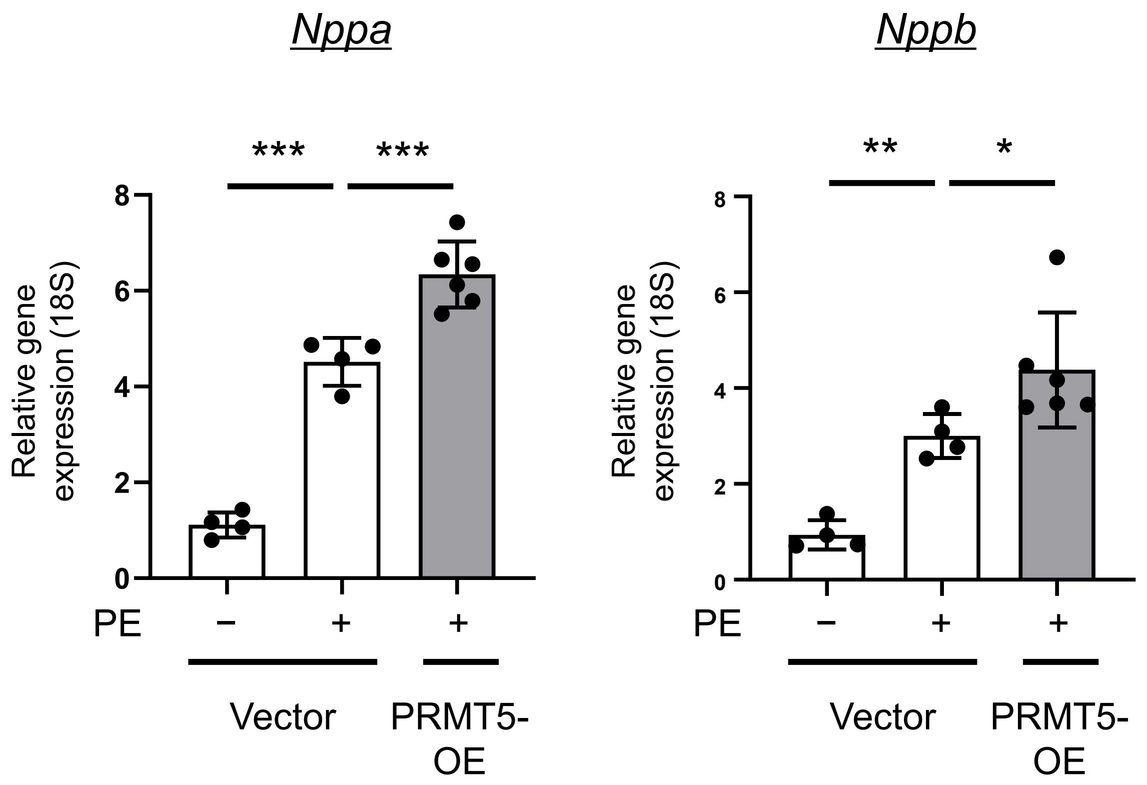

Supplementary figure 4
The lentiviral transduction of FLAG-PRMT5 promotes hypertrophic gene expression in cultured cardiomyocytes. Values are presented as mean ± SD (n = 4–6). Data are analyzed using one-way ANOVA, followed by Dunnett's multiple comparison tests versus the PE-treated group. * $p < 0.05$, ** $p < 0.01$, *** $p < 0.001$.
